# TPL2 mediates IL-17R signaling in neuroinflammation

**DOI:** 10.18632/oncotarget.4888

**Published:** 2015-07-17

**Authors:** Yichuan Xiao, Shao-Cong Sun

**Affiliations:** Institute of Health Sciences, Shanghai Institutes for Biological Sciences, Chinese Academy of Sciences/Shanghai Jiao Tong University School of Medicine, Shanghai, China; Department of Immunology, The University of Texas MD Anderson Cancer Center, Houston TX, USA. The University of Texas Graduate School of Biomedical Sciences, Houston, TX, USA

**Keywords:** Immunology and Microbiology Section, Immune response, Immunity

TPL2 (tumor progression locus 2), also known as COT and MAP3K8, was initially identified as an oncogene and is now known to play important roles in the regulation of both tumor growth and inflammation [1]. In unstimulated cells, TPL2 associates with the NF-κB1 precursor protein p105 and the ubiquitin-binding protein ABIN-2 (A20-binding inhibitor of NF-κB 2) to form a complex that serves to maintain the protein stability of TPL2 and inhibits its kinase function. The death domain (DD) of p105 directly interacts with the kinase domain (KD) of TPL2, thereby inhibiting the access of TPL2 by its substrate, MEK1. TPL2 can be activated by ligands of various toll-like receptors (TLRs) and proinflammatory cytokines via a mechanism that requires the IκB kinase (IKK). IKK phosphorylates p105 and induces p105 degradation, which releases TPL2 and allows TPL2 to phosphorylate MEK1 and activate the downstream kinases ERK1 and ERK2 (ERK1/2) [1]. Previous studies by using primary cells from Tpl2-deficient mice revealed an essential role for TPL2 in mediating activation of the MEK1-ERK1/2 signaling pathway in myeloid cells (dendritic cells, macrophages) and in embryonic fibroblasts following stimulation by TLR ligands, TNF-α, and IL-1β.

We and others have recently identified TPL2 as a crucial signaling factor mediating neuroinflammation in an animal model of multiple sclerosis (MS): experimental autoimmune encephalomyelitis (EAE) [2, 3]. MS is a chronic disease of the central nervous system (CNS), characterized by inflammation, demyelination, and axonal damage [4]. Accumulating studies suggest the crucial involvement of IL-17-producing Th17 subset of inflammatory T cells in the induction of EAE [5, 6]. Genome-wide association studies and functional analyses have further linked IL-17 to the pathogenesis of both MS and other autoimmune diseases. In addition, IL-17 receptor (IL-17R) signaling in neuroectoderm-derived CNS-resident cells, particularly astrocytes and NG2^+^ glial cells, plays a crucial role in mediating EAE pathogenesis. In our recent study, we have found that TPL2-deficient mice are refractory to EAE induction due to attenuated leukocyte recruitment to the CNS [2]. Although TPL2 is dispensable for Th17 cell differentiation and function, TPL2 functions in nonhematopoitic cells to mediate the induction of EAE [2, 3]. *In vitro* experiments demonstrate that TPL2 functions in astrocytes to mediate IL-17- stimulated expression of chemokines and proinflammatory cytokines in addition to its role in regulating TLR-stimulated gene expression in microglia.

**Figure 1:**
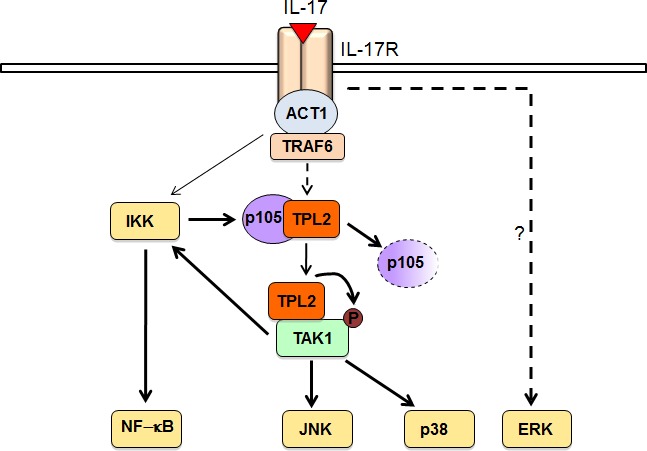
A model of TPL2 signaling function in the IL-17R pathway IL-17 stimulate initial weak activation of IKK, which stimulates the signaling function of TPL2 via mediating phosphorylation-dependent p105 degradation. Liberated TPL2 binds to and phosphorylates TAK1, triggering TAK1 catalytic activity and the activation of JNK and p38. TAK1 also activates IKK, which serves as a positive feedback mechanism that is required for optimal activation of the TPL2-TAK1 signaling axis. IL-17-stimulated ERK activation is independent of TPL2.

How the IL-17R signal is transduced to the various downstream pathways, particularly the MAP kinases (MAPKs) and IKK pathways, has not been fully elucidated. The protein kinase TAK1 has been shown to be important for IL-17-stimulated activation of IKK and its downstream transcription factor NF-κB, although how the IL-17R signal activates TAK1 has remained unclear [7]. We have obtained genetic and biochemical evidence that TPL2 acts by connecting the IL-17R signal to the activation of TAK1 [2]. TPL2 directly interacts with TAK1 and activates TAK1 via phosphorylation. In the IL-17R signaling pathway, TAK1 is crucial, although not absolutely essential, for activation of both IKK and the MAPKs JNK and p38. The Tpl2- and TAK1-deficient cells retain the ability for a low level of IKK activation upon IL-17 stimulation, suggesting the existence of a TPL2/TAK1-independent pathway of IKK activation. This latter pathway appears to be important for initiating the TPL2/TAK1 signaling axis, since IKK ablation abolishes IL-17- stimulated activation of TPL2 and TAK1. Based on these data, we propose a model for how TPL2 regulates IL-17R signaling (Figure 1). In response to IL-17 stimulation, IKK becomes activated at a moderate level and mediates phosphorylation-dependent degradation of p105, thereby causing the liberation of TPL2 and allowing TPL2 to associate with its substrate TAK1. Upon activation by TPL2, TAK1 activates the downstream kinases JNK and p38 and promotes activation of IKK in a positive-feedback loop. Thus, our data reveal a previously unappreciated facet of IL-17R signaling that involves a dynamic mechanism mediating the activation and function of the TPL2-TAK1 axis.

Our results also raise some outstanding questions. Firstly, TPL2 is best known as a kinase that mediates MEK1/ERK activation by TLRs and TNFR. However, despite the common downstream pathways shared by the TLRs, TNFR and IL-17R, we have found that TPL2 is completely dispensable for ERK1/2 activation by the IL- 17R, thus suggesting fundamental differences in the signal transduction mediated by the TLRs/TNFR and IL-17R. Precisely how ERK1/2 is activated in IL-17R signaling pathway needs to be further investigated. Secondly, the precise CNS cell type in which TPL2 functions to regulate EAE pathogenesis remains to be determined. Thirdly, the important role of TPL2 in mediating IL-17R signaling raises the question of whether it also regulates the inflammatory responses in other autoimmune diseases. Finally, our data implicate TPL2 as a potential therapeutic target for the treatment of MS. Future studies with small molecule inhibitors of TPL2 will further assess this possibility.
